# Quantification of 24,25‐Dihydroxyvitamin D_3_ in Serum Using LC–MS/MS With Derivatization and Lipid‐Removal Filtration

**DOI:** 10.1155/ianc/5736140

**Published:** 2026-02-24

**Authors:** Marta Studecká, Eliška Fousková, Josefa Provalilová, Roman Viták, Zdeněk Tůma, Michal Jirásko, Markéta Králová, Kateřina Oulehle, Jan Vachek, Ladislav Pecen, Radek Kučera, Richard Pikner

**Affiliations:** ^1^ Department of Clinical Biochemistry and Bone Metabolism, Klatovska Hospital, Klatovy, 33901, Czech Republic; ^2^ Department of Pharmacology and Toxicology, Faculty of Medicine in Pilsen, Charles University, Pilsen, 32300, Czech Republic, cuni.cz; ^3^ Biomedical Center, Faculty of Medicine in Pilsen, Charles University, Pilsen, 32300, Czech Republic, cuni.cz; ^4^ Department of Immunochemistry Diagnostics, University Hospital and Faculty of Medicine in Pilsen, Charles University, Pilsen, 32300, Czech Republic, cuni.cz; ^5^ Department of Internal Medicine, Klatovska Hospital, Klatovy, 33901, Czech Republic; ^6^ Department of Nephrology, 1^st^ Faculty of Medicine and General University Hospital, Charles University, Prague, 12808, Czech Republic, cuni.cz; ^7^ Department of Clinical Biochemistry and Hematology, Faculty of Medicine Pilsen, Charles University, Pilsen, 32300, Czech Republic, cuni.cz

## Abstract

Accurate assessment of vitamin D metabolism is crucial not only for the diagnosis and treatment of disorders related to bone health and calcium homeostasis but also for understanding its broader physiological roles in immunity, cellular differentiation, cardiovascular regulation, and endocrine function. Although 25‐hydroxyvitamin D_3_ (25(OH)D_3_) is routinely measured in clinical practice, the low‐abundance metabolite 24,25‐dihydroxyvitamin D_3_ (24,25(OH)_2_D_3_) provides complementary insight into vitamin D catabolism. Reduced or undetectable 24,25(OH)_2_D_3_ levels may signal impaired CYP24A1 function or insufficient conversion of 25(OH)D_3_ to maintain appropriate intracellular concentrations of biologically active 1,25(OH)_2_D_3_. However, quantification of 24,25(OH)_2_D_3_ remains analytically challenging and requires highly selective and sensitive liquid chromatography–tandem mass spectrometry (LC–MS/MS) methodology. In‐house developed LC–MS/MS approaches are being increasingly employed, offering greater specificity and sensitivity over conventional immunoassays, allowing accurate detection of low‐abundance vitamin D metabolites. Additionally, when interpreted together with 25(OH)D_3_, 24,25(OH)_2_D_3_ allows the calculation of the vitamin D metabolite ratio (VMR), which offers a more accurate assessment of vitamin D sufficiency and catabolism. The aim of this study was to develop and validate a robust LC–MS/MS method using dynamic multiple reaction monitoring (dMRM) and sample derivatization for quantifying 24,25(OH)_2_D_3_ in human serum. The method described was evaluated according to EMA guidelines and DEQAS controls. The matrix effect was minimized through lipid‐removal filtration. The assay demonstrated excellent linearity (*R* ^2^ = 0.9982), intra‐ and inter‐assay precision below 14%, and LOQ of 0.64 ng/mL. Recovery from DEQAS samples ranged from 80% to 118%, with a matrix‐induced ion enhancement of ∼17%. Additionally, 4‐phenyl‐1,2,4‐triazoline‐3,5‐dione (PTAD) derivatization enhanced the sensitivity 100‐fold. This highly sensitive LC–MS/MS method is suitable for clinical and research laboratories equipped with an electrospray ionization (ESI) source. Precise quantification of 24,25(OH)_2_D_3_ can complement routine 25(OH)D_3_ analysis, support VMR determination, and serve as a reliable biomarker for disorders associated with altered vitamin D metabolism.

## 1. Introduction

Vitamin D is a secosteroid pro‐hormone essential for proper bone and muscle development [1, 2]. It also modulates several physiological processes, including innate and adaptive immunity, cell growth and differentiation, cardiovascular function, and hormonal actions [3–5]. Its deficiency has become a global issue that is affecting individuals across all ages and genders. The primary causes include limited sun exposure and insufficient dietary intake of vitamin D from food sources [6].

Vitamin D encompasses a group of six compounds (D_2_–D_7_), with D_3_ (cholecalciferol) and D_2_ (ergocalciferol) being the most significant for humans. Vitamin D_3_ is synthesized in the skin through UVB irradiation of 7‐dehydrocholesterol and can also be obtained from animal‐based foods like fish, eggs, and fortified milk. In contrast, vitamin D_2_ is derived exclusively from plants, fungi, and yeasts [7, 8].

In the human body, vitamin D undergoes multiple hydroxylation processes, each facilitated by specific enzymes. The first hydroxylation occurs in the liver, primarily mediated by the key enzyme CYP2R1, which converts vitamin D to 25(OH)D. This compound is the major circulating form of vitamin D. The second hydroxylation step, known as 1α‐hydroxylation, takes place mainly in the kidneys. In this process, 25(OH)D is converted into the hormonally active form of vitamin D, 1,25(OH)_2_D. This metabolite binds to the vitamin D receptor and, therefore, plays a significant role in the biological effects of vitamin D [9, 10]. Finally, hydroxylation, which is mediated by the enzyme CYP24A1, is responsible for the multistep catabolism of both 25(OH)D and 1,25(OH)_2_D, converting them into 24,25(OH)_2_D and 1,24,25(OH)_3_D [11]. In healthy individuals without calcium supplementation, the metabolite 24,25(OH)_2_D has a circulating half‐life of ∼7 days and is present at concentrations of ng/mL, typically in a range of 1–5 ng/mL [12, 13].

Usually, vitamin D sufficiency is determined by measuring serum concentrations of 25(OH)D [14, 15]. However, there is an increasing interest in the determination of other vitamin D metabolites, especially in 24,25(OH)_2_D_3_, which is a major dihydroxyvitamin D metabolite in a serum used as a catabolism marker, for example, for kidney disease [13, 16]. Furthermore, emerging evidence suggests that a more accurate marker may be the vitamin D metabolite ratio (VMR), calculated as a ratio of 24,25(OH)_2_D to 25(OH)D [17, 18]. This ratio reflects the activity of CYP24A1, which, with increased enzymatic activity, raises the levels of 24,25(OH)_2_D and, consequently, the VMR [19]. Measuring the VMR provides additional insights into vitamin D catabolism and can predict individual responses to vitamin D supplementation [18]. Therefore, a precise method for quantification of 24,25(OH)_2_D_3_ can serve as a useful tool to complement data obtained from the determination of routinely analyzed 25(OH)D, provide necessary results in clinical practice to determine diseases arising from vitamin D metabolism deficiencies, and help to personalize the treatment for individual patients.

The methods used for measuring vitamin D can vary significantly. Immunoassays are commonly employed due to their low cost and accessibility. However, immunoassays often suffer from issues with cross‐reactivity, which prevents them from accurately distinguishing between different forms of vitamin D [5, 20]. On the other hand, liquid chromatography coupled with mass spectrometry (LC–MS) allows for precise differentiation between these forms, which is why it is the gold standard for vitamin D measurement [21, 22]. Despite the high acquisition costs, the need for specialized equipment, and the optimization of sample preparation, LC–MS‐based assays can provide the data necessary for detailed insight into vitamin D metabolism.

This article describes an in‐house LC–MS/MS method for determining 24,25(OH)_2_D_3_ along with the description of method development with high precision and method validation. This approach enables accurate quantification of 24,25(OH)_2_D_3_ and offers additional context for interpreting vitamin D metabolism and its clinical implications.

## 2. Materials and Methods

### 2.1. Reagents

For method development and validation, a labeled standard ^2^H_6_‐24,25(OH)_2_D_3_ (≥ 97%, IsoScience) and a native form 24,25(OH)_2_D_3_ (IsoScience) were used. Both standards were supplied in liquid form, dissolved in ethanol, with a concentration of 0.1071 mg/mL for ^2^H_6_‐24,25(OH)_2_D_3_ and 100 mg/mL for the native form 24,25(OH)_2_D_3_.

Vitamin D‐free serum (Mass Spect Gold Human Serum, Ultra‐Low Vitamin D, GoldenWest Biologicals, USA) was used to prepare calibration and control samples. A calibration curve was established with concentrations ranging from 0.5 to 16 ng/mL, while the validation was conducted in the range of 0.5–8 ng/mL. A solution of ^2^H_6_‐24,25(OH)_2_D_3_ in acetonitrile (ACN) at a concentration of 10 ng/mL served as the internal standard.

All solvents used during the development of our method were LC/MS‐grade, including methanol and ACN (Honeywell), and water from an OmniaTap system (StakPure) was equipped with a final LC/MS purification filter. Formic acid (Sigma Aldrich) and ammonium fluoride (Sigma Aldrich) were employed to adjust the mobile phase. Sample preparation and purification were carried out using Captiva EMR‐Lipid columns (Agilent). Additionally, 4‐phenyl‐1,2,4‐triazoline‐3,5‐dione (PTAD) (> 97%, Sigma Aldrich) was used to prepare the derivatization solution, which consisted of ACN containing 1 mg/mL PTAD.

### 2.2. Sample Preparation

Prior to analysis, serum samples were stored at −80°C, thawed in a water bath at 30°C, and vortexed for 10 s. Following this step, protein precipitation was carried out at room temperature, where 100 μL of serum sample was treated with 400 μL of ACN and 4% zinc sulfate (ZnSO_4_) solution. To purify the samples, 100 μL of each sample and 400 μL of internal standard solution were applied to the Captiva EMR‐Lipid Column. The mixture was filtered into Eppendorf tubes under vacuum conditions. Next, 400 μL of pure ACN was added to the column, which was filtered again under vacuum into the Eppendorf tubes to release the analyte adsorbed on the column’s sorbent. The samples were evaporated using a heated concentrator under an N_2_ stream until complete evaporation occurred. After complete evaporation, samples were derivatized for 1 h at room temperature using 100 μL of PTAD derivatization reagent (1 mg/mL in ACN). Following derivatization, the samples were transferred to measurement vials and directly injected in a 3‐μL volume into the LC–MS/MS system.

### 2.3. LC/MS Conditions

Identification and quantification of analytes were conducted using the Agilent 6495 Triple Quadrupole LC/MS System with an electrospray ionization (ESI) source. Chromatographic separation was achieved by using the 1290 Infinity II LC with an Agilent RRHD Eclipse Plus C18 column (2.1 × 50 mm, particle size 1.8 μm). The separation was carried out at 30°C, with a 3‐μL injection volume. Prior to analysis, samples were stored in the autosampler at 15°C.

### 2.4. Gradient Elution

A gradient elution was applied using the following:

• Mobile Phase A: water modified with formic acid 0.01% (v/v) and 1 mM ammonium fluoride.
• Mobile Phase B: methanol.

### 2.5. Gradient Details

• 0 min: 50% A, 50% B.
• 1.5 min: 25% A, 75% B.
• 3.0 min: 0% A, 100% B.
• 4.0 min: 0% A, 100% B.
• 4.1 min: 50% A, 50% B.

The total analysis time was 5 min, with a mobile phase flow rate of 0.4 mL/min.

### 2.6. Ionization and Detection

Samples were ionized in positive ESI mode. Optimal signal conditions included the following:

• Source temperature: 225°C.
• Gas temperature: 100°C.
• Gas flow: 13 L/min.
• Nebulizer pressure: 45 psi.
• Sheath gas temperature: 370°C.
• Sheath gas flow: 11 L/min.
• Capillary voltage: +3500 V.

Detection was performed in the dynamic multiple reaction monitoring (dMRM) mode with a cycle time of 500 ms (needle flush 3 s). The dwell time for the individual transitions was set to 95.56 ms per cycle. The retention time window (RT window) was set to 1.03 min.

### 2.7. Data Processing

Data acquisition and processing were performed using the following Agilent software tools:

• Agilent MassHunter Qualitative Analysis 10.0.
• Agilent MassHunter Quantitative Analysis (for QQQ).
• Agilent MassHunter Data Acquisition.

This method ensures robust and reliable quantification of analytes with precise chromatographic separation and sensitive detection.

## 3. Results

### 3.1. Method Development

**FIGURE 1:**
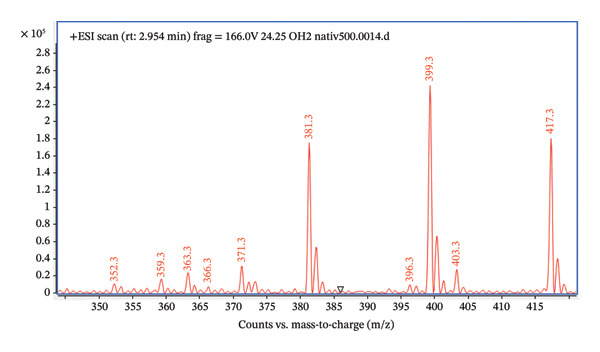
Full scan of native 24,25(OH)_2_D_3_.

For the method development, we first used the default parameters using an ESI ion source. We performed a full scan for a pure standard with a concentration of 5 μg/mL (Figure 1), mobile phase methanol/water (50:50), flow rate of 0.4 mL/min, and analysis time of 5 min. The analyte was ionized in positive mode, resulting in [M + H]^+^. The signal m/z 417.3 corresponds to the protonated molecule of 24,25(OH)_2_D_3_.

**FIGURE 3:**
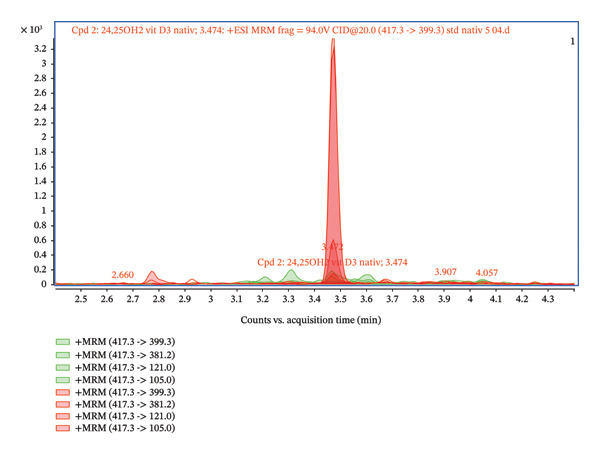
Comparison of the 24,25(OH)_2_D_3_ standard 5 ng/mL (red) and the spiked 5 ng/mL serum after protein precipitation (green).

**FIGURE 4:**
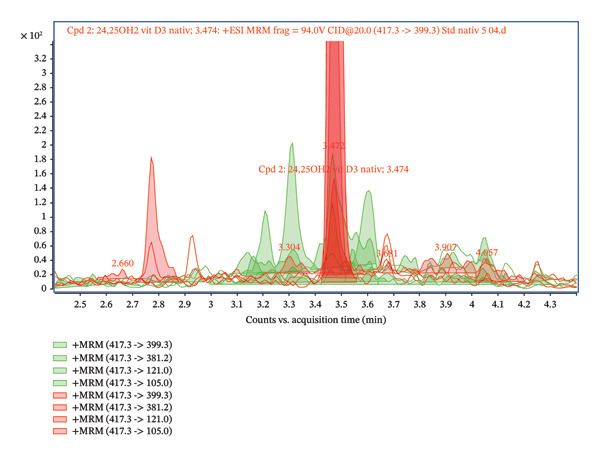
Comparison of the 24,25(OH)_2_D_3_ standard 5 ng/mL (red) and the spiked 5 ng/mL serum after protein precipitation (green)—detailed.

Furthermore, the chromatogram shown in Figure 2 presents the results for several transitions analyzed in the dMRM mode. This approach enables the selective detection of molecules according to their characteristic fragments. Individual transitions refer to various combinations of precursor ions (m/z) and their corresponding product ions generated during fragmentation in the collision chamber. The transition m/z = 417.3 ⟶ 121.0 shows the highest intensity, which provides a dominant and well‐defined chromatographic peak, and this fragment is therefore suitable for quantification. The transitions 417.3 ⟶ 105.0, 417.3 ⟶ 381.2, and 417.3 ⟶ 399.3 show lower but reproducible intensities and serve as qualification transitions to confirm the identity of the analyte. For the analysis, we used an ESI ion source system, which is sensitive to matrix effects (MEs), and requires thorough sample preparation. The primary issues in this context stem from the presence of unremoved phospholipids, lipids, and other matrix contaminants, which can negatively impact the ionization and fragmentation of the target analyte. Our first step involved employing a common protein precipitation method using ACN and a 4% ZnSO_4_ solution. The result of the precipitation is presented in Figures 3 and 4, comparing the signal of a pure 24,25(OH)_2_D_3_ standard at a concentration of 5 ng/mL with that of a spiked serum at the same concentration after protein precipitation. The chromatogram in Figures 3 and 4 confirms that the pure standard produces a sharp and clearly defined peak at a retention time of 3.47 min. Compared to the native standard at the same concentration (5 ng/mL), the signal of the spiked serum is noticeably more diffuse and displays significant background noise. This indicates the persistent influence of the ME. Thus, protein precipitation alone is insufficient for sample preparation intended for LC–MS/MS analysis of 24,25(OH)_2_D_3_.

FIGURE 2(a) Overlay chromatograms of the dMRM transition 417.3 ⟶ 399.3 of native 24,25(OH)2D3, showing the retention time at approximately 2.96 min. (b) Extracted ion chromatograms of individual dMRM transitions of the precursor ion m/z 417.3 (417.3 ⟶ 121.0, 417.3 ⟶ 105.0, 417.3 ⟶ 381.2, and 417.3 ⟶ 399.3). The transition 417.3 ⟶ 121.0 exhibits the highest signal intensity and was selected as the quantifier, while the remaining transitions serve as qualifiers. All transitions co‐elute at approximately 2.95–2.97 min, confirming their origin from the same analyte.

**Figure:**
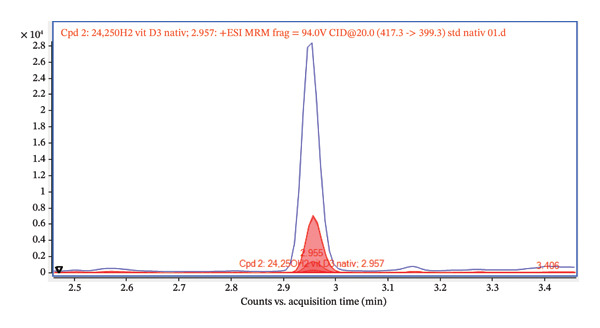
(a)

**Figure:**
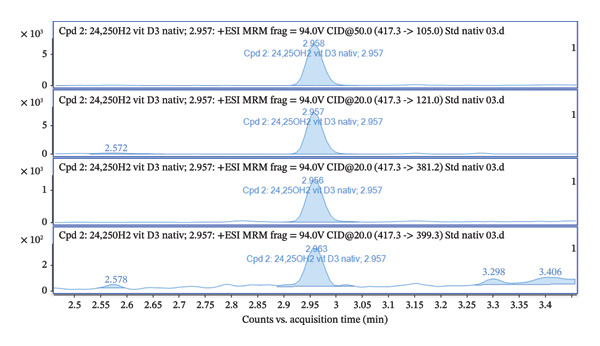
(b)

**FIGURE 5:**
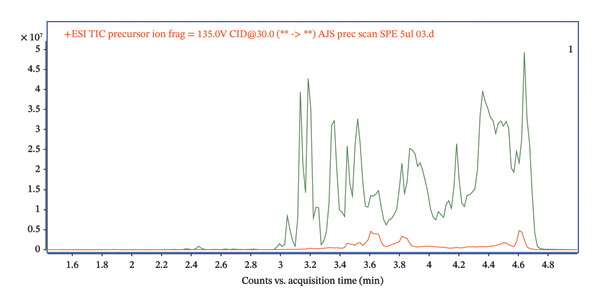
Comparison of precursor scan (m/z = 184) for protein precipitation procedure (green) and purification of spiked serum by lipid‐removal filtration (red).

Phospholipids are abundant in plasma, and their ability to suppress ESI ionization contributes to the ME [23]. The presence of phospholipids can be monitored by precursor ion scanning of m/z 184 [24]. In our case, there was a strong signal at m/z 184 throughout the whole chromatogram, suggesting that phospholipids can contribute to the ME in spiked plasma (green, Figure 5).

To minimize ME and ensure the most accurate measurement of target analytes, samples were prepared prior to analysis by lipid‐removal filtration through Captiva EMR‐Lipid columns (Agilent) that contain a specific sorbent. These columns are specifically designed to effectively remove lipids, phospholipids, and other matrix interferents without compromising the analyte. The lipid‐removal filtration process relies on size exclusion and hydrophobic interactions, which allow long aliphatic lipid chains to be selectively captured on the sorbent’s surface. Additionally, the columns feature a specialized frit that captures the solvent, facilitating efficient protein precipitation during lipid‐removal filtration.

A comparison of precursor ion scanning of m/z 184 in the spiked serum and the spiked serum treated by lipid‐removal filtration through Captiva EMR‐Lipid columns showed depletion of the signal of this phospholipid marker. The presence of lipids can significantly interfere with the analysis, especially affecting the measurement of target analytes (green). In contrast, the red curve, representing the sample postlipid‐removal filtration, exhibits a dramatic decrease in signal intensity, which confirms the column’s effectiveness in removing phospholipids (Figure 5).

The chromatogram in Figure 5 clearly demonstrates that the lipid removal step is essential for increasing the accuracy and reproducibility of the analysis while minimizing the ME that could adversely affect the detection and quantification of the target analyte. This approach fosters cleaner separations and ensures that analytical results remain unaltered by lipid matrix interferents.

**FIGURE 6:**
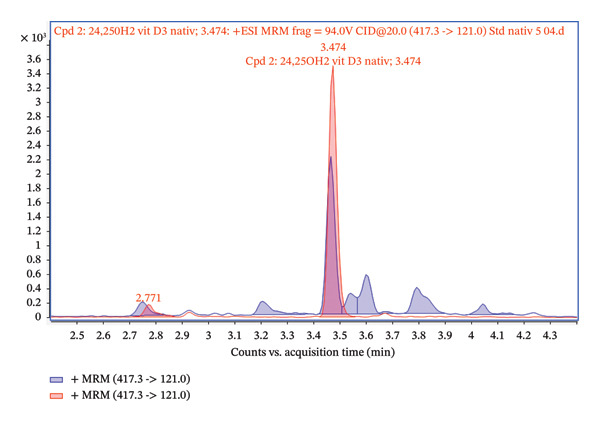
Recovery of 24,25(OH)_2_D_3_ standard (5 ng/mL) (red) and spiked serum (5 ng/mL) after lipid‐removal filtration (blue).

Figure 6 shows the chromatogram illustrating the precursor ion transition of 417.3 ⟶ 121.0, which was selected as a quantifier for analyzing 24,25(OH)_2_D_3_. Two sample variants of the analyte with the same concentration (5 ng/mL) are compared: a pure standard of 24,25(OH)_2_D_3_ and a spiked serum sample containing 24,25(OH)_2_D_3_ after purification through lipid‐removal column filtration. The main peak (red), occurring at a retention time of 3.47 min, corresponds to the target analyte, 24,25(OH)_2_D_3_. This peak is sharp, narrow, and well‐defined, indicating high sample purity. In contrast, the peak in the spiked serum sample is broader and less pronounced, suggesting that the ME is present, even after lipid‐removal filtration. This observation implies that residual matrix components may still affect the ionization of the target analyte, likely due to its low abundance in the serum. However, the signal intensity of the spiked serum closely corresponds to the native standard. This demonstrates that the lipid‐removal filtration step preserves the analyte, with minimal loss during sample preparation.

Figure 7 illustrates a comparison of two chromatograms of spiked serum samples (5 ng/mL). One chromatogram shows the sample after lipid‐removal filtration without derivatization (green), while the other displays the spiked serum sample after derivatization with the PTAD reagent (blue). Derivatization with PTAD enhances the sensitivity and selectivity of analyte detection, particularly for vitamin D and its derivatives. Derivatization is used in LC–MS analysis of vitamin D to increase the ionization efficiency, making the compounds easier to detect and increasing sensitivity, especially for low‐abundance metabolites [25]. Figure 7 demonstrates that derivatization significantly reduces interference from matrix components. The peak of the derivatized sample is clearly dominant and lacks side signals near the retention time, indicating that the derivatization step enhances selectivity and accuracy in analysis. Additionally, after derivatization, the signal intensity increased 100‐fold compared to the spiked serum sample that had only undergone lipid‐removal filtration. This experiment clearly illustrates the advantages of derivatization in improving detection quality, especially within complex biological matrices like the serum. The final ionic transitions after derivatization are summarized in Table 1.

FIGURE 7Chromatogram of (a) 24,25(OH)_2_D_3_ spiked serum (5 ng/mL) without derivatization (green) and (b) after PTAD derivatization (blue).

**Figure:**
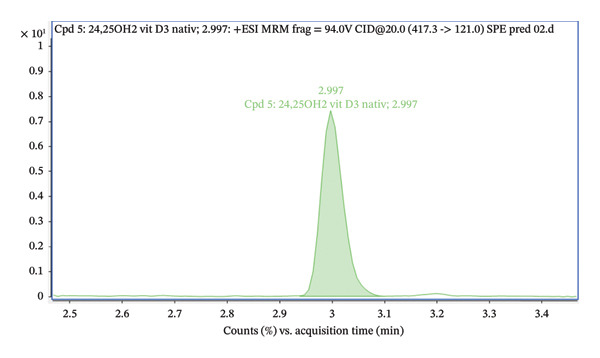
(a)

**Figure:**
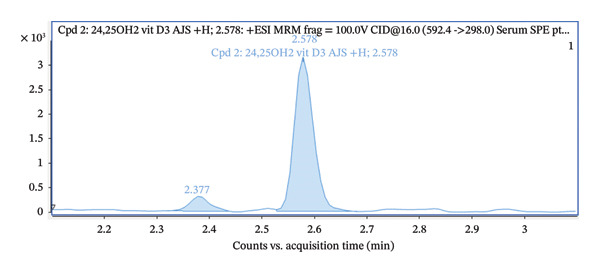
(b)

**TABLE 1:** Ionic transitions after derivatization with PTAD for native form 24,25(OH)_2_D_3_ and labeled standard H2 6‐24,25(OH)_2_D_3._

Compound	Precursor ion	Product ion	Ret. time (min)	CE (V)
PTAD‐24,25(OH)_2_D_3_	592.4	298	2.6	20
PTAD‐24,25(OH)_2_D_3_	592.4	280	2.6	36
PTAD‐24,25(OH)_2_D_3_	592.4	161	2.6	48
PTAD‐d6‐24,25(OH)_2_D_3_	598.3	580.4	2.6	8
PTAD‐d6‐24,25(OH)_2_D_3_	598.3	298	2.6	20

The transition (592.4 ⟶ 298) was used for quantification, while the qualifier transitions (592.4 ⟶ 280 and 592.4 ⟶ 161) were used for identity confirmation (Figure 8). Ion ratios for transitions of qualifiers are shown in Table 2. Acceptance criteria for ion ratios were ± 20% of the mean value obtained from calibration standards. The precursor ion 592.4 corresponds to the 24,25(OH)_2_D_3_ after PTAD derivatization.

FIGURE 8(a) Total ion chromatogram acquired in the dMRM mode for PTAD‐derivatized 24,25(OH)2D3, showing the dominant chromatographic peak eluting at approximately 2.58 min. (b) Extracted ion chromatograms of individual dMRM transitions of the precursor ion m/z 592.4 corresponding to PTAD‐derivatized 24,25(OH)2D3. The transition 592.4 ⟶ 298.0 exhibits the highest signal intensity and was selected as the quantifier, while the transitions 592.4 ⟶ 280.0 and 592.4 ⟶ 161.0 were used as qualifier ions for identity confirmation. All monitored transitions co‐elute at approximately 2.58 min, confirming consistent fragmentation and chromatographic behavior of the derivatized analyte.

**Figure:**
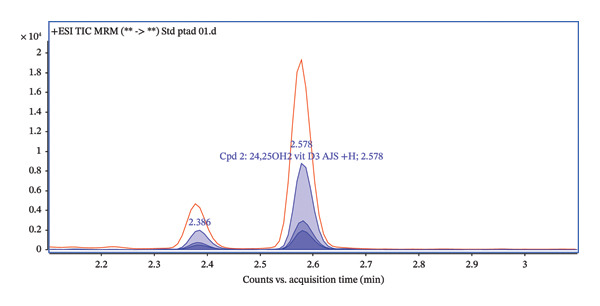
(a)

**Figure:**
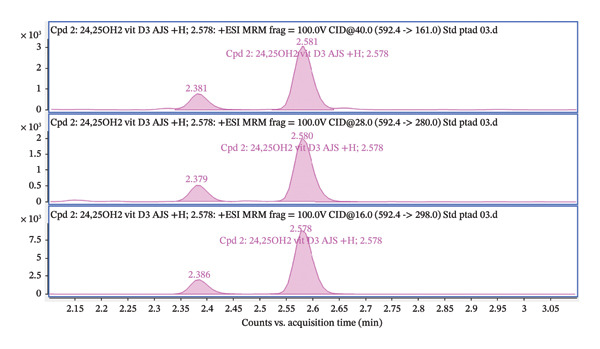
(b)

**TABLE 2:** Ion ratios for transitions of 592.4 ‐> 280.0 and 592.4 ‐> 161.0 represented by mean value ± SD obtained from three repetitions of calibration standard samples.

Concentration (ng/mL)	Qualifier (592.4 ‐> 280.0) ion ratio mean ± SD	Qualifier (592.4 ‐> 161.0) ion ratio mean ± SD
0.5	35.1000 ± 8.0225	29.9667 ± 0.6351
1	31.8667 ± 0.9238	26.3333 ± 0.9238
2	30.2000 ± 0.3464	30.4000 ± 1.5588
4	30.2000 ± 1.5588	29.5000 ± 1.0392
8	30.6000 ± 0.1732	31.0000 ± 0.0000
16	29.9667 ± 3.2332	31.8000 ± 1.9053

### 3.2. Method Validation

To validate our methodology, the following analytical parameters were measured: linearity, limit of quantification (LOQ) and limit of detection (LOD), precision (assessed by both inter‐assay and intra‐assay), and accuracy (DEQAS). Additionally, we assessed the presence of the ME, the autosampler stability of the prepared samples, and carry‐over.

**FIGURE 9:**
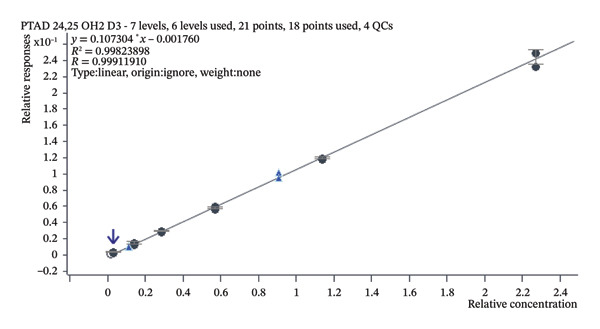
Calibration curve indicating the relationship between the relative concentration of PTAD 24,25(OH)_2_D_3_ standard (ng/mL) and relative response. *R* ^2^ corresponds to a 0.99824 value.

The linearity of our measurements was verified using a series of six levels within the concentration range of 0.5–16 ng/mL; all concentration levels were measured in triplicate. The calibration curve was constructed using a linear regression model obtained by plotting the peak area ratios of analytes/IS against their nominal concentrations, with no weighting applied (weight: none). The analyte response ratio to IS was used for regression analysis. A correlation coefficient (*R* ^2^) of 0.9982 indicated satisfactory linearity. In Figure 9, a calibration curve is shown illustrating the relationship between relative response and relative concentration of PTAD 24,25(OH)_2_D_3_ correlated to PTAD ^2^H_6_‐24,25(OH)_2_D_3_ IS.

The LOQ was calculated using Agilent MassHunter Quantitative Analysis software (for QQQ). The LOQ and LOD were established from ten replicate measurements at the lowest point of the calibration curve (0.5 ng/mL) and were defined on the basis of the signal‐to‐noise (S/N) ratio. LOQ resulted in a value of 0.64 ng/mL, and LOD was calculated as 0.19 ng/mL (CV 8.8%).

The precision was assessed through inter‐assay and intra‐assay evaluations. Following EMA guidelines, inter‐assay precision was evaluated by analyzing six concentration levels: 0.5 ng/mL (lower limit of quantification, LLOQ), 1, 2, 4, 6, and 8 ng/mL. All measurements were conducted over a period of 10 days, with inter‐assay precision ranging from 4.3% to 13.8%. Intra‐assay precision was analyzed using six concentration levels: 0.5, 1, 2, 4, 6, and 8 ng/mL. Five repeated measurements were taken in a single day for each concentration level, resulting in intra‐assay precision ranging from 3.6% to 11.8%. The results are shown in Table 3.

**TABLE 3:** The method precision represented by CV (%) results from intra‐assay and inter‐assay for 24,25(OH)_2_D_3_ analysis.

Concentration (ng/mL)	Arithmetic average (ng/mL)	SD	Intra‐assay precision (CV %)	Arithmetic average (ng/mL)	SD	Inter‐assay precision (CV %)
0.5	0.5061	0.0601	11.8	0.5162	0.0712	13.8
1	0.8882	0.0989	11.1	0.9739	0.1268	13.0
2	1.9413	0.1066	10.6	1.8547	0.2042	11.0
4	3.5982	0.1320	3.6	3.6320	0.3012	8.2
6	6.1862	0.2361	3.8	6.1863	0.2671	4.3
8	8.1187	0.4703	5.8	8.1188	0.4531	5.6

The method’s accuracy was verified using DEQAS samples collected during October 2023, April 2024, and October 2024. The results are presented in Table 4.

**TABLE 4:** Comparison of measured 24,25(OH)_2_D_3_ sample concentrations with DEQAS reference values and recovery rate.

	10/2023				04/2024				10/2024		
	DEQAS (ng/mL)	Our value (ng/mL)	Recovery (%)		DEQAS (ng/mL)	Our value (ng/mL)	Recovery (%)		DEQAS (ng/mL)	Our value (ng/mL)	Recovery (%)
641	2.60	2.45	94.23	651	1.10	1.08	98.18	661	0.90	0.80	88.89
642	0.40	0.39	97.25	652	2.40	2.44	101.67	662	1.80	1.54	85.56
643	0.80	0.67	83.75	653	0.80	0.64	80.00	663	2.10	2.48	118.09
644	2.00	2.07	103.50	654	1.50	1.50	100.00	664	1.10	1.28	116.36
645	0.50	0.43	86.00	655	1.60	1.60	100.00	665	1.20	1.40	116.67

#### 3.2.1. Statistical Analysis for DEQAS Accuracy Method

**FIGURE 10:**
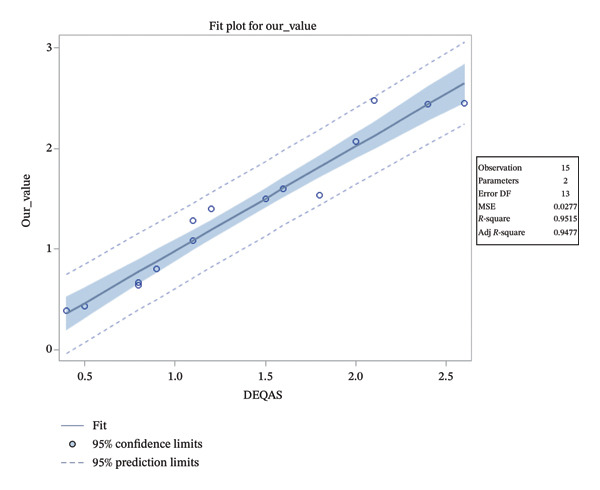
Linear regression plot comparing our measured values and DEQAS reference samples (both in ng/mL) assessing the method’s accuracy. The solid line represents the fitted regression model; the shaded band indicates the 95% confidence interval for the mean fit, and the dashed lines show the 95% prediction limits. Number of samples (*n* = 15). The model demonstrates a high degree of linearity (*R* ^2^ = 0.9515).

The bias between our method and reference DEQAS samples was assessed. The mean bias was −0.00200, 95% confidence interval (CI; −0.09218, 0.08818), and the median bias was −0.01, 95% distribution‐free CI (−0.13, 0.07), where measured values were between 0.39 and 2.60. We can conclude that the bias is very close to zero. The relatively wide 95% CIs reflect the limited number of samples (*n* = 15). An ideal recovery is 100%. Observed mean recovery is 98.02662%, 95% CI (91.30162%, 104.75162%), which is very close to the ideal value. The linear regression model demonstrates that our value is a linear function of DEQAS, which leads to a nonsignificant intercept of −0.06018, 95% CI (−0.27660, 0.15625), and a significant slope of 1.04196, 95% CI (0.90096, 1.18296; *p*‐value < 0.0001). These results are very close to the ideal values, where the intercept is 0.00000 and the slope is 1.00000, indicating good agreement with the reference method. These results are represented by a linear regression plot shown in Figure 10.

**FIGURE 11:**
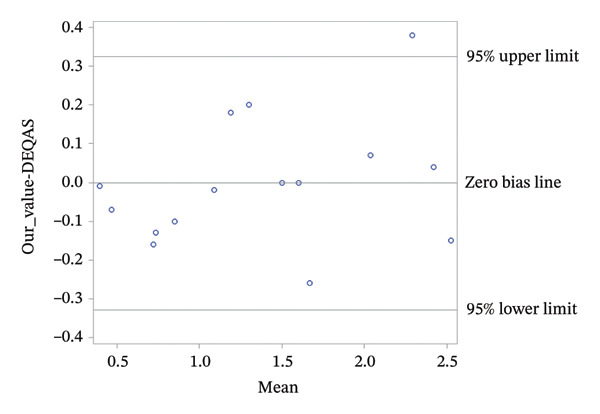
Bland–Altman plot comparing our method with DEQAS reference values. The central horizontal line represents the mean bias. The upper and lower horizontal lines indicate the ± 1.96 standard deviation limits of agreement. The plot shows homogenous differences across the measurement range, with only one value outside the upper limit. All remaining points lie within the expected limits.

The Bland–Altman plot shows homogeneity of the differences with only one value falling outside the ±1.96 standard deviations interval (DEQAS 2.40 ng/mL, our value 2.44 ng/mL, recovery 101.67%), which is consistent with the expectation that most of the individual differences are expected to fall within this range. These results are shown in Figure 11.

Finally, the statistical analysis demonstrates that results obtained from our developed methodology are in strong agreement with standard reference measurements (DEQAS). The results indicate that the proposed approach for quantifying 24,25(OH)_2_D_3_ is suitable for clinical application with high accuracy.

**FIGURE 12:**
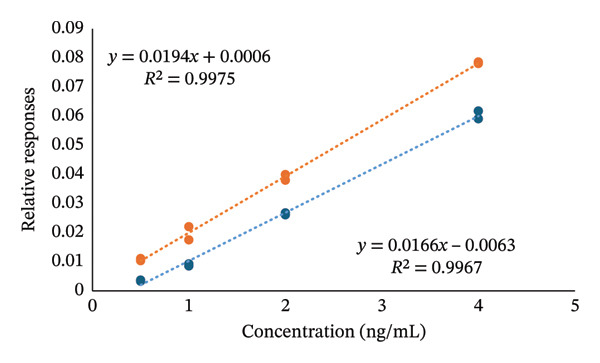
The orange curve represents serum samples, and the blue curve represents 24,25(OH)_2_D_3_ standard in LC/MS‐grade water. Both calibration curves demonstrate a high degree of linearity (*R* ^2^ = 0.9975 and 0.9967).

ME was calculated from slopes of two calibration curves. One set of samples was prepared in LC/MS‐grade water without any matrix (blue) at concentrations of 0.5, 1, 2, and 4 ng/mL, while another set was prepared using vitamin D‐free serum as a matrix (orange). Both calibration curves, which illustrate the relationship between relative response and the concentration of 24,25(OH)_2_D_3_ (ng/mL), are shown in Figure 12. Addition of matrix led to ion enhancement; the resulting ME was 16.9%.

(1) % ME=SlopematrixSlopesolvent×100−100.

The stability was tested on samples prepared as a part of the inter‐assay precision, which were measured after 24 h and stored in an autosampler at 15°C. The recovery rates from the two repeated measurements for concentrations 0.5–6 ng/mL ranged from 79.13% to 116.38%. The results for the stability test of 24,25(OH)_2_D_3_ conducted after 24 h and storage in an autosampler at 15°C are a part of the Supporting Information (available here).

**FIGURE 13:**
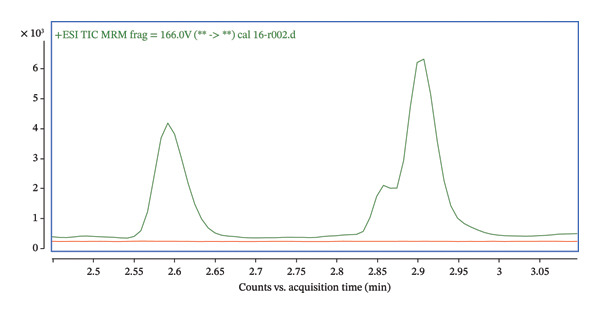
Chromatogram illustrating the carry‐over data representing the calibrator at 16 ng/mL (green) followed by the subsequent blank injection (red). The absence of a detectable peak in the blank confirms no significant carry‐over.

Additionally, the carry‐over was evaluated to exclude the presence of residual sample from the previous injection during the analysis. Results are shown in Figure 13, representing the chromatogram showing the calibrator at 16 ng/mL (green) and the measured blank (red). The absence of a detectable peak in the blank line confirms that no significant carry‐over occurred under the applied analysis conditions.

## 4. Discussion

Quantification of the metabolite 24,25(OH)_2_D_3_ has gained increasing clinical relevance as a biomarker for vitamin D catabolism deficiencies, offering additional information beyond 25(OH)D, which is being routinely analyzed. Determination of 25(OH)D is routine in general clinical laboratories and has been previously discussed elsewhere [26–30]. However, it may not be sufficient, and there is increasing evidence to support the determination of 24,25(OH)_2_D_3_ and subsequent calculation of VMR to improve the assessment of vitamin D status [18, 31]. The determination of low‐abundance vitamin D metabolites in complex biological matrices such as serum is particularly challenging. Although antibody‐based immunoassays are widely used in vitamin D determination due to their high‐throughput analysis with minimal sample preparation, they are nevertheless largely limited by analytical specificity. This limitation is due to cross‐reactions with other structurally related metabolites resulting from poor antibody specificity [10]. For this reason, LC–MS/MS methodologies have become the preferred analytical approach, as they enable reliable quantification of multiple vitamin D metabolites present in trace concentrations [32]. The challenges for efficient LC–MS/MS protocol are mainly in managing ionization efficiency and combating the matrix interference while addressing rapid and precise analysis performance. Our study addresses these analytical challenges by combining PTAD derivatization, lipid‐removal filtration, and dMRM detection in a single workflow suitable for clinical laboratory application. Currently, ESI and atmospheric pressure chemical ionization (APCI) are used in LC–MS/MS methods for vitamin D and its metabolites. ESI is more selective for polar compounds, while APCI is better suited for less polar compounds. Vitamin D and its metabolites naturally have low ionization efficiency under typical ESI conditions; therefore, APCI is the preferred ionization method for these molecules. However, derivatization with PTAD increases its polarity and significantly increases its ionization efficiency in ESI, leading to an increase in signal intensity during mass spectrometry analysis. Moreover, derivatization supports the analysis by shifting the mass to higher m/z values with less isobaric noise and by providing specific fragmentation patterns for MS/MS [25]. In our approach, the PTAD derivatization enabled higher molecule polarity and increased ionization efficiency by 100‐fold.

Moreover, the use of Captiva EMR‐Lipid cartridges effectively removed phospholipids, which are among the main contributors to ion interference, as confirmed by precursor ion scanning at m/z 184. Importantly, the application of dMRM allowed transitions to be monitored solely within a narrow retention window of 1.03 min corresponding to the elution of derivatized 24,25(OH)_2_D_3_. This increased dwell time per transition (95.56 ms per cycle), provided more data points across the chromatographic peak, and yielded substantially improved signal intensity and S/N ratio compared with conventional MRM. At the same time, dMRM minimized interfering signals outside the retention region and improved the selectivity of the measurement for the low‐abundance vitamin D metabolite.

Importantly, the proposed method shows strong agreement with external reference values (DEQAS). Recovery rates range from 80% to 118%, a mean bias is close to zero (−0.00200), and only one value in the Bland–Altman analysis is outside the ±1.96 standard deviation interval, confirming our methodology is free of systematic bias and fits well within the analysis. Therefore, we can conclude that our methodology strongly agrees with the international standards of vitamin D measurements, providing us the external control as a validation of methodological accuracy.

Several previously described LC–MS/MS methods have achieved reliable detection of 24,25(OH)_2_D_3_ and have also utilized PTAD [12, 33–36] or DPTAD [37] derivatization to enhance ionization efficiency. However, their protocols typically involve more extensive sample processing or lack lipid‐removal filtration, which can compromise reproducibility in complex biological matrices, and they usually present longer analysis times. Our approach relies on the use of Captiva EMR‐Lipid columns to remove phospholipids, which significantly improved the S/N ratio and reduced ME, as confirmed by precursor ion scanning (m/z = 184). Furthermore, our method enables rapid analysis, with a total run time of 5 min. This represents an advantage over several previously published in‐house protocols, which require a longer process. Shorter run time is highly desirable in routine or high‐throughput analyses, especially in clinical practice, to achieve high analytical efficiency. For example, Satoh et al. developed the method for the measurement of 24,25(OH)_2_D_3_, where they reported a 5.5 min analysis time [37]. Additionally, Zelzer et al. compared two LC–MS/MS strategies for quantifying 24,25(OH)_2_D_3_. In their ESI‐based approach, they reported a 17 min run time in total, therefore prolonging the whole process [38]. In contrast, our method reduces the total analysis time while maintaining the accuracy and precision.

## 5. Conclusion

In summary, the described method combines high analytical performance with clinical relevance, supporting its potential role in advancing the diagnostic evaluation of vitamin D‐related disorders and enzymatic deficiencies. Although our method is focused on the targeted quantification of 24,25(OH)_2_D_3_, it is compatible with expansion to broader analytical panels. Its minimized analysis time, simple derivatization protocol, and compatibility with standard triple quadrupole equipment make it an efficient and scalable tool for both clinical and translational research focused on vitamin D metabolism. Our method offers validated analysis and shows strong agreement with external quality assessment samples from DEQAS. This confirms the suitability of the method for clinical laboratories with access to LC–MS/MS technology.

## Funding

This study was supported by​ the Ministry of Health, Czech Republic, for the conceptual development of research organization (Faculty Hospital in Pilsen‐FNPl, 00669806), BBMRI‐CZ: Biobank Net‐Work—a versatile platform for the research of the etiopathogenesis of diseases CZ.02.1.01/0.0/0.0/16_013/000167 and LM2015089 and by the “Cooperatio” Program, Research Area Pharmaceutical Sciences.

## Conflicts of Interest

The authors declare no conflicts of interest.

## Supporting Information

Additional supporting information can be found online in the Supporting Information section.

## Supporting information


**Supporting Information 1** Supporting Information 1—This document provides a description of the LC–MS/MS methodology for the 25(OH)D_3_ metabolite, including sample preparation and measurement conditions, together with method validation data. Validation results include inter‐ and intra‐assay precision and accuracy assessed by comparison of analyzed 25(OH)D_3_ with DEQAS performance data.

**Supporting Information 2** Supporting Information 2—This table presents autosampler stability data for 24,25(OH)_2_D_3_ following 24‐h storage at 15°C after sample preparation. Obtained concentrations before and after the timepoint are reported along with the recovery rates.

**Supporting Information 3** Supporting Information 3—This document contains detailed statistical calculations supporting the method comparison analysis, including bias estimation, Bland–Altman analysis, and regression statistics. The tables provide transparency on deriving the reported statistical data.

**Supporting Information 4** Supporting Information 4—This Excel file includes raw calibration data from three independent calibration measurements for 24,25(OH)_2_D_3_. The table reports measured values and corresponding ion ratios used for calibration assessment.

**Supporting Information 5** Supporting Information 5—This file shows 4 representative chromatograms presenting ion ratios for the analyte 24,25(OH)_2_D_3_ and ^2^H_6_‐24,25(OH)_2_D_3_ serving as an internal standard. This Supporting Information serves as complementary data for Table 2 in the manuscript.

## Data Availability

Data are available from the corresponding author upon reasonable request.
