# Supplementary material for: Quantification of 24,25‐Dihydroxyvitamin D3 in Serum Using LC–MS/MS With Derivatization and Lipid‐Removal Filtration
Source: Int J Anal Chem. 2026 Feb 24;2026:5736140. doi: 10.1155/ianc/5736140 (PMC12930099; doi:10.1155/ianc/5736140)
Supplement: Supplementary file 1 — Supporting Information 1 Supporting Information 1—This document provides a description of the LC–MS/MS methodology for the 25(OH)D3 metabolite, including sample preparation and measurement conditions, together with method validation data. Validation results include inter‐ and intra‐assay precision and accuracy assessed by comparison of analyzed 25(OH)D3 with DEQAS performance data. [file IANC-2026-5736140-s004.docx]

# **Materials and Methods for the Determination of 25(OH)D_3_ Metabolite by LC-MS/MS**

## **Reagents**

For method development and validation, a labeled standard, ²H_3_-25(OH)D₃ (99.45%, IsoScience), and the native form, 25(OH)D₃ (99.49%, IsoScience), were used. Standards were supplied in liquid form, dissolved in ethanol, with a concentration of 50 mg/mL for ²H_3_-25(OH)D₃ and 0.05449 mg/mL for the native form 25(OH)D₃.

Vitamin D-free serum (Mass Spect Gold Human Serum, Ultra-Low Vitamin D, GoldenWest Biologicals, USA) was used to prepare calibration and control samples. The method calibration and validation were conducted in the range of 4 to 60 ng/mL. Solution of ²H_3_-25(OH)D₃ in acetonitrile at a concentration of 10 ng/mL served as internal standard.

All solvents used during the development of our method were LC/MS-grade, including methanol and acetonitrile (Honeywell), and water from an OmniaTap system (StakPure) was equipped with a final LC/MS purification filter. Formic acid (Sigma Aldrich) and 1 mM ammonium fluoride (Sigma Aldrich) were employed to adjust the mobile phase. Sample preparation and purification were carried out using Captiva EMR-Lipid columns (Agilent).

## **Sample Preparation**

Prior to analysis, serum samples were stored at -80 °C, thawed in a water bath at 30 °C, and vortexed for 10 seconds. Following this step, protein precipitation was carried out at room temperature, where 100 μL of serum sample was treated with 400 μL of acetonitrile and 4% zinc sulfate (ZnSO_4_) solution. To purify the samples, 100 µL of each sample and 400 µL of internal standard solution were applied to the Captiva EMR-Lipid Column. The mixture was filtered into Eppendorf tubes under vacuum conditions. Next, 400 µL of pure acetonitrile was added to the column, which was filtered again under vacuum into the Eppendorf tubes to release the analyte adsorbed on the column's sorbent. Following lipid-removal filtration, 25(OH)D_3_ samples were injected in a 5 μL volume into LC-MS/MS.

## **LC/MS Conditions**

Identification and quantification of analytes were performed on the Agilent 6495 Triple Quadrupole LC/MS coupled to a 1290 Infinity II LC and ESI ion source (Agilent). Chromatographic separation was carried out on Poroshell 120 PFP column (4.6 × 50 mm, particle size 2.7 µm, Agilent). The separation was conducted at 20 °C, with an injection volume of 5 µL. Prior to analysis, samples were stored in the autosampler at 15 °C.

### **Gradient Elution**

A gradient elution was applied using:

- **Mobile Phase A:** Water modified with formic acid 0.01 % (v/v) and 1 mM ammonium fluoride.
- **Mobile Phase B:** Methanol.

### **Gradient Details:**

- 0 min: 35% A, 65% B.
- 1.0 min: 25% A, 75% B.
- 6.0 min: 20% A, 80% B.
- 6.5 min: 0% A, 100% B.
- 8.5 min: 35% A, 65% B.

The total analysis time was 10 minutes, with a mobile phase flow rate of 0.4 mL/min.

### **Ionization and Detection**

Samples were ionized using ESI in positive mode. Optimal signal was achieved under the following conditions:

- Source temperature: **225 °C**.
- Gas temperature: **200 °C**.
- Gas flow: **20 L/min**.
- Nebulizer pressure: **45 psi**.
- Sheath gas temperature: **400 °C**.
- Sheath gas flow: **12 L/min**.
- Capillary voltage: **+3500 V**.

Detection was performed in the MRM mode, the total cycle time was 10 min. The dwell time for the individual transitions was set to 753.7 ms per cycle.

### **Data Processing**

Data acquisition and processing were performed using the following Agilent software tools:

- **Agilent MassHunter Qualitative Analysis 10.0.**
- **Agilent MassHunter Quantitative Analysis (for QQQ).**
- **Agilent MassHunter Data Acquisition.**

This method ensures robust and reliable quantification of the analyte with precise chromatographic separation and sensitive detection.

# **Results**

### **Performance Characteristics for 25(OH)D_3_**

*Table 1: The method precision represented by CV (%) results from intra-assay for 25(OH)D₃ analysis.*

| **concentration [ng/mL]** | **arithmetic average [ng/mL]** | **SD** | **precision %** | **intra-assay precision [CV%]** |
| --- | --- | --- | --- | --- |
| 4 | 3,27 | 0,26 | 0,901 | 8,03 |
| 12 | 11,35 | 0,36 | 0,945 | 3,21 |
| 40 | 39,72 | 2,67 | 0,929 | 6,73 |
| 60 | 68,01 | 3,22 | 0,946 | 4,74 |

*Table 2: The method precision represented by CV (%) results from inter-assay for 25(OH)D₃ analysis.*

| **ng/ml** | **arithmetic average [ng/mL]** | **SD** | **precision %** | **CV %** |
| --- | --- | --- | --- | --- |
| 4 | 3,82 | 0,71 | 0,953 | 18,77 |
| 12 | 11,87 | 1,04 | 0,989 | 8,82 |
| 40 | 38,19 | 3,84 | 0,952 | 10,05 |
| 60 | 60,02 | 5,70 | 0,999 | 9,51 |

*Table 3: Comparison of measured 25(OH)D₃ sample concentrations with DEQAS reference values and recovery rate for accuracy assessment.*

| **DEQAS 2/2024** | **[ng/mL]** | **DEQAS [ng/mL]** | **recovery %** |
| --- | --- | --- | --- |
| **646** | 34,10 | 37,24 | 91,60 |
| **647** | 24,16 | 23,72 | 101,80 |
| **648** | 28,03 | 27,00 | 103,80 |
| **649** | 17,30 | 17,20 | 100,80 |
| **650** | 11,04 | 11,50 | 96,10 |
