# Supplementary material for: Quantification of 24,25‐Dihydroxyvitamin D3 in Serum Using LC–MS/MS With Derivatization and Lipid‐Removal Filtration
Source: Int J Anal Chem. 2026 Feb 24;2026:5736140. doi: 10.1155/ianc/5736140 (PMC12930099; doi:10.1155/ianc/5736140)
Supplement: Supplementary file 2 — Supporting Information 2 Supporting Information 2—This table presents autosampler stability data for 24,25(OH)2D3 following 24‐h storage at 15°C after sample preparation. Obtained concentrations before and after the timepoint are reported along with the recovery rates. [file IANC-2026-5736140-s005.docx]

Table: 24,25(OH)_2_D_3_ sample stability data obtained after 24 hours after preparation and storage in an autosampler at 15 °C. Data shows measured metabolites concentrations (ng/mL) before and after 24-hour timepoint with recovery rate.

| **concentration [ng/mL]** | **28.5.2024** | **29.5.2024** | **recovery [%]** | **2.6.2024** | **3.6.2024** | **recovery [%]** |
| --- | --- | --- | --- | --- | --- | --- |
| 0.5 | 0.5392 | 0.4659 | 86.41 | 0.4385 | 0.4702 | 107.23 |
| 0.5 | 0.4783 | 0.4798 | 100.31 | 0.5472 | 0.5229 | 95.56 |
| 1 | 1.1759 | 1.1066 | 94.11 | 0.9660 | 0.9953 | 103.03 |
| 1 | 1.2593 | 0.9965 | 79.13 | 0.9908 | 0.8957 | 90.40 |
| 2 | 1.8971 | 1.9700 | 103.84 | 1.8739 | 2.1808 | 116.38 |
| 2 | 1.8293 | 1.9503 | 106.61 | 1.9867 | 2.1595 | 108.70 |
| 4 | 3.9183 | 3.8127 | 97.30 | 3.8377 | 3.6472 | 95.04 |
| 4 | 3.9996 | 3.7312 | 93.29 | 3.9702 | 4.0757 | 102.66 |
| 6 | 6.1312 | 6.0533 | 98.73 | 5.7060 | 5.4718 | 95.90 |
| 6 | 5.9576 | 6.1286 | 102.87 | 6.0017 | 5.9035 | 98.36 |
