# Supplementary material for: Quantification of 24,25‐Dihydroxyvitamin D3 in Serum Using LC–MS/MS With Derivatization and Lipid‐Removal Filtration
Source: Int J Anal Chem. 2026 Feb 24;2026:5736140. doi: 10.1155/ianc/5736140 (PMC12930099; doi:10.1155/ianc/5736140)

| ***Variable: bias = our_value - DEQAS*** |
| --- |

| **Basic Statistical Measures** | | | |
| --- | --- | --- | --- |
| **Location** | | **Variability** | |
| **Mean** | -0.00200 | **Std Deviation** | 0.16284 |
| **Median** | -0.01000 | **Variance** | 0.02652 |
| **Mode** | 0.00000 | **Range** | 0.64000 |

| **Basic Confidence Limits Assuming Normality** | | | |
| --- | --- | --- | --- |
| **Parameter** | **Estimate** | **95% Confidence Limits** | |
| **Mean** | -0.00200 | -0.09218 | 0.08818 |
| **Std Deviation** | 0.16284 | 0.11922 | 0.25682 |
| **Variance** | 0.02652 | 0.01421 | 0.06595 |

| **Tests for Location: Mu0=0** | | | | |
| --- | --- | --- | --- | --- |
| **Test** | **Statistic** | | **p Value** | |
| **Student's t** | **t** | -0.04757 | **p-value** | 0.9627 |
| **Sign** | **M** | -1.5 | **p-value** | 0.5811 |
| **Signed Rank** | **S** | -4 | **p-value** | 0.7998 |

There was no evidence of a systematic deviation from zero bias.

| **Location Counts: Mu0=0.00** | |
| --- | --- |
| **Count** | **Value** |
| **Num Obs > Mu0** | 5 |
| **Num Obs < Mu0** | 8 |

| **Quantiles (Definition 5)** | | | | | | | | |
| --- | --- | --- | --- | --- | --- | --- | --- | --- |
| **Level** | **Quantile** |  | | | | **Order Statistics** | | |
|  |  | **95% Confidence Limits Assuming Normality** | | **95% Confidence Limits Distribution Free** | | **LCL Rank** | **UCL Rank** | **Coverage** |
| **100% Max** | 0.38 |  |  |  |  |  |  |  |
| **99%** | 0.38 | 0.25392 | 0.62045 | . | . | . | . | . |
| **95%** | 0.38 | 0.16506 | 0.45353 | 0.18 | 0.38 | 13 | 15 | 50.05 |
| **90%** | 0.20 | 0.11534 | 0.36672 | 0.07 | 0.38 | 12 | 15 | 73.86 |
| **75% Q3** | 0.07 | 0.02530 | 0.22766 | -0.01 | 0.38 | 8 | 15 | 96.93 |
| **50% Median** | -0.01 | -0.09218 | 0.08818 | -0.13 | 0.07 | 4 | 12 | 96.48 |
| **25% Q1** | -0.13 | -0.23166 | -0.02930 | -0.26 | -0.01 | 1 | 8 | 96.93 |
| **10%** | -0.16 | -0.37072 | -0.11934 | -0.26 | -0.13 | 1 | 4 | 73.86 |
| **5%** | -0.26 | -0.45753 | -0.16906 | -0.26 | -0.15 | 1 | 3 | 50.05 |
| **1%** | -0.26 | -0.62445 | -0.25792 | . | . | . | . | . |
| **0% Min** | -0.26 |  |  |  |  |  |  |  |

| ***Variable: recovery = (our_value - DEQAS) * 100 %*** |
| --- |

| **Basic Statistical Measures** | | | |
| --- | --- | --- | --- |
| **Location** | | **Variability** | |
| **Mean** | 98.0266 | **Std Deviation** | 12.14378 |
| **Median** | 98.1818 | **Variance** | 147.47137 |
| **Mode** | 100.0000 | **Range** | 38.09524 |
|  |  | **Interquartile Range** | 17.50000 |

| **Basic Confidence Limits Assuming Normality** | | | |
| --- | --- | --- | --- |
| **Parameter** | **Estimate** | **95% Confidence Limits** | |
| **Mean** | 98.02662 | 91.30162 | 104.75162 |
| **Std Deviation** | 12.14378 | 8.89078 | 19.15194 |
| **Variance** | 147.47137 | 79.04603 | 366.79688 |

| **Quantiles (Definition 5)** | | | | | | | | |
| --- | --- | --- | --- | --- | --- | --- | --- | --- |
| **Level** | **Quantile** |  | | | | **Order Statistics** | | |
|  |  | **95% Confidence Limits Assuming Normality** | | **95% Confidence Limits Distribution Free** | | **LCL Rank** | **UCL Rank** | **Coverage** |
| **100% Max** | 118.0952 |  |  |  |  |  |  |  |
| **99%** | 118.0952 | 117.1120 | 144.4454 | . | . | . | . | . |
| **95%** | 118.0952 | 110.4851 | 131.9977 | 116.3636 | 118.0952 | 13 | 15 | 50.05 |
| **90%** | 116.6667 | 106.7776 | 125.5235 | 103.5000 | 118.0952 | 12 | 15 | 73.86 |
| **75% Q3** | 103.5000 | 100.0622 | 115.1532 | 98.1818 | 118.0952 | 8 | 15 | 96.93 |
| **50% Median** | 98.1818 | 91.3016 | 104.7516 | 86.0000 | 103.5000 | 4 | 12 | 96.48 |
| **25% Q1** | 86.0000 | 80.9000 | 95.9910 | 80.0000 | 98.1818 | 1 | 8 | 96.93 |
| **10%** | 83.7500 | 70.5298 | 89.2757 | 80.0000 | 86.0000 | 1 | 4 | 73.86 |
| **5%** | 80.0000 | 64.0556 | 85.5682 | 80.0000 | 85.5556 | 1 | 3 | 50.05 |
| **1%** | 80.0000 | 51.6078 | 78.9412 | . | . | . | . | . |
| **0% Min** | 80.0000 |  |  |  |  |  |  |  |

| ***Regression … model our_value - linear function of DEQAS*** |
| --- |

| **Number of Observations Read** | 15 |
| --- | --- |
| **Number of Observations Used** | 15 |

| **Parameter Estimates** | | | | | |
| --- | --- | --- | --- | --- | --- |
| **Variable** | **DF** | **Parameter Estimate** | **Standard Error** | **t Value** | **p-value** |
|  |  |  |  |  |  |
| **Intercept** | 1 | -0.06018 | 0.10018 | -0.60 | 0.5584 |
| **DEQAS** | 1 | 1.04196 | 0.06527 | 15.96 | <.0001 |

| **Parameter Confidence Intervals** | | | | | |
| --- | --- | --- | --- | --- | --- |
| **Variable** | **DF** |  | | | |
|  |  | **95% Confidence Limits** | | **Heteroscedasticity Consistent 95% Confidence Limits** | |
| **Intercept** | 1 | -0.27660 | 0.15625 | -0.21142 | 0.09107 |
| **DEQAS** | 1 | 0.90096 | 1.18296 | 0.91200 | 1.17192 |


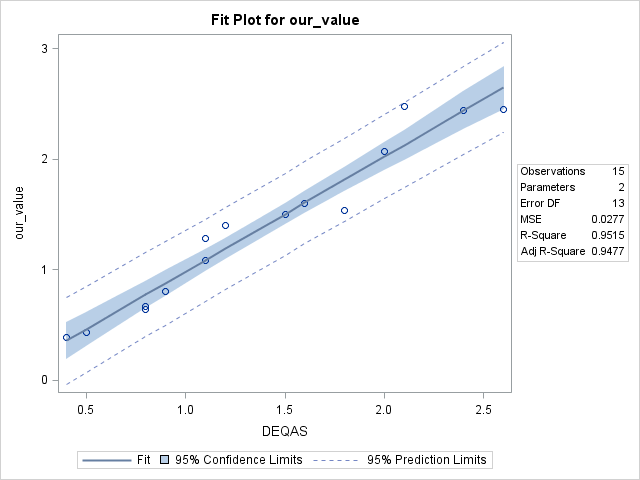


***Bland-Altman Plot***


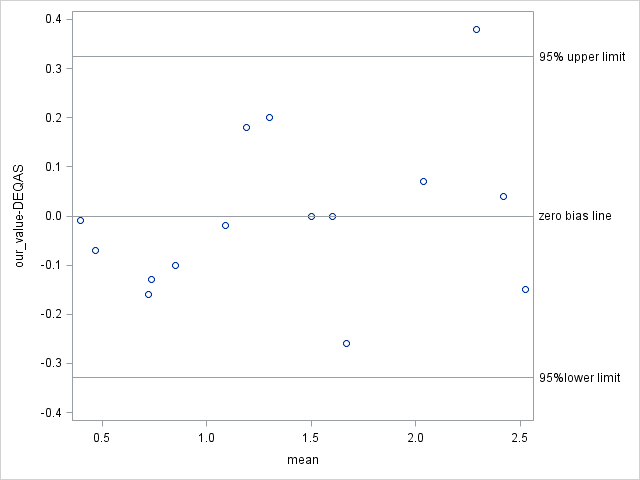

Supplement: Supplementary file 3 — Supporting Information 3 Supporting Information 3—This document contains detailed statistical calculations supporting the method comparison analysis, including bias estimation, Bland–Altman analysis, and regression statistics. The tables provide transparency on deriving the reported statistical data. [file IANC-2026-5736140-s002.docx]
