# Supplementary material for: Quantification of 24,25‐Dihydroxyvitamin D3 in Serum Using LC–MS/MS With Derivatization and Lipid‐Removal Filtration
Source: Int J Anal Chem. 2026 Feb 24;2026:5736140. doi: 10.1155/ianc/5736140 (PMC12930099; doi:10.1155/ianc/5736140)

**Chromatograms for 24,25(OH)_2_D_3_ sample and corresponding ion ratios**


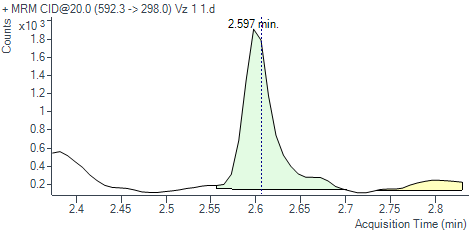


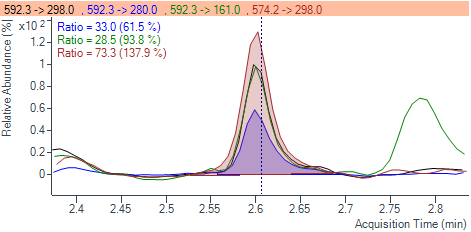


**Chromatograms for** **^2^H_6_-24,25(OH)_2_D_3_ internal standard sample and corresponding ion ratios**


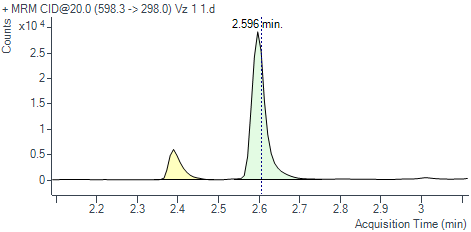


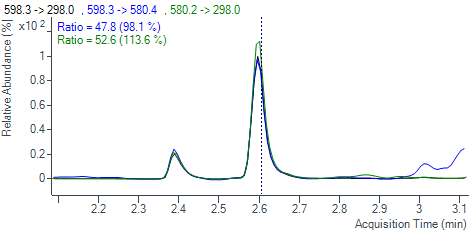

Supplement: Supplementary file 5 — Supporting Information 5 Supporting Information 5—This file shows 4 representative chromatograms presenting ion ratios for the analyte 24,25(OH)2D3 and 2H6‐24,25(OH)2D3 serving as an internal standard. This Supporting Information serves as complementary data for Table 2 in the manuscript. [file IANC-2026-5736140-s001.docx]
